# Hand-hygiene compliance by hospital staff and incidence of health-care-associated infections, Finland

**DOI:** 10.2471/BLT.19.247494

**Published:** 2020-05-26

**Authors:** Helena Ojanperä, Outi I Kanste, Hannu Syrjala

**Affiliations:** a Research Unit of Nursing Science and Health Management, University of Oulu, Aapistie 5A, 2 krs 90220 Oulu, Finland.; b Department of Infection Control, Oulu University Hospital, Oulu, Finland.

## Abstract

**Objective:**

To determine changes in hand-hygiene compliance after the introduction of direct observation of hand-hygiene practice for doctors and nurses, and evaluate the relationship between the changes and the incidence of health-care-associated infections.

**Methods:**

We conducted an internal audit survey in a tertiary-care hospital in Finland from 2013 to 2018. Infection-control link nurses observed hand-hygiene practices based on the World Health Organization’s strategy for hand hygiene. We calculated hand-hygiene compliance as the number of observations where necessary hand-hygiene was practised divided by the total number of observations where hand hygiene was needed. We determined the incidence of health-care-associated infections using a semi-automated electronic incidence surveillance programme. We calculated the Pearson correlation coefficient (*r*) to evaluate the relationship between the incidence of health-care-associated infections and compliance with hand hygiene.

**Findings:**

The link nurses made 52 115 hand-hygiene observations between 2013 and 2018. Annual hand-hygiene compliance increased significantly from 76.4% (2762/3617) in 2013 to 88.5% (9034/10 211) in 2018 (*P* < 0.0001). Over the same time, the number of health-care-associated infections decreased from 2012 to 1831, and their incidence per 1000 patient-days fell from 14.0 to 11.7 (*P* < 0.0001). We found a weak but statistically significant negative correlation between the monthly incidence of health-care-associated infections and hand-hygiene compliance (r = −0.48; *P* < 0.001).

**Conclusion:**

The compliance of doctors and nurses with hand-hygiene practices improved with direct observation and feedback, and this change was associated with a decrease in the incidence of health-care-associated infections. Further studies are needed to evaluate the contribution of hand hygiene to reducing health-care-associated infections.

## Résumé

**Objectif:**

Identifier les changements dans l'application des mesures d'hygiène des mains après l'introduction d'un système d'observation directe des pratiques en la matière chez les médecins et infirmiers. Évaluer le rapport entre ces changements et le nombre d'infections liées aux soins de santé.

**Méthodes:**

Nous avons effectué un audit interne au sein d'un hôpital de soins tertiaires en Finlande entre 2013 et 2018. Les infirmières chargées du contrôle des infections ont jugé les pratiques d'hygiène en se basant sur la stratégie d'hygiène des mains de l'Organisation mondiale de la Santé. Nous avons défini dans quelle mesure le personnel respectait les consignes d'hygiène des mains en divisant le nombre de cas où le niveau d'hygiène requis était respecté par le nombre total de cas où l'hygiène des mains était nécessaire. Nous avons également déterminé le nombre d'infections liées aux soins de santé à l'aide d'un programme de surveillance électronique semi-automatique. Enfin, nous avons calculé le coefficient de corrélation de Pearson (*r*) afin d'évaluer le rapport entre le nombre d'infections liées aux soins de santé et le respect de l'hygiène des mains.

**Résultats:**

Les infirmières ont observé 52 115 cas d'hygiène des mains entre 2013 et 2018. La moyenne annuelle du respect des mesures a considérablement augmenté, passant de 76,4% (2762/3617) en 2013 à 88,5% (9034/10 211) en 2018 (*P* < 0,0001). Durant la même période, le nombre d'infections liées aux soins de santé a diminué, passant de 2012 à 1831, et leur fréquence sur 1000 jours-patients a chuté, de 14,0 à 11,7 (*P* < 0,0001). Nous avons détecté une corrélation négative faible mais statistiquement significative entre le taux d'incidence mensuel d'infections liées aux soins de santé et le respect de l'hygiène des mains (r = −0,48; *P* < 0,001).

**Conclusion:**

Les observations directes et retours ont encouragé les médecins et infirmiers à mieux respecter les consignes d'hygiène des mains. Ce changement a entraîné une baisse du nombre d'infections liées aux soins de santé. D'autres études sont cependant nécessaires pour évaluer l'impact de l'hygiène des mains sur la diminution des infections liées aux soins de santé.

## Resumen

**Objetivo:**

Determinar los cambios del cumplimiento de la higiene de las manos tras las observaciones directas en las prácticas de higiene de las manos que realizan los médicos y las enfermeras, además de evaluar la relación entre los cambios y la incidencia de las infecciones relacionadas con la atención médica.

**Métodos:**

Se realizó una encuesta de auditoría interna en un hospital de atención terciaria en Finlandia de 2013 a 2018. Las enfermeras de control de infecciones observaron las prácticas de higiene de las manos según la estrategia de la Organización Mundial de la Salud para la higiene de las manos. Se estimó que el cumplimiento de la higiene de las manos equivalía a dividir el número de las observaciones en las que era necesario practicar la higiene de las manos por el número total de observaciones en las que se requería la higiene de las manos. Se determinó la incidencia de las infecciones relacionadas con la atención médica mediante un programa semiautomatizado y electrónico de vigilancia de la incidencia. Se calculó la correlación de Pearson (*r*) para evaluar la relación entre la incidencia de las infecciones relacionadas con la atención médica y el cumplimiento de la higiene de las manos.

**Resultados:**

Las enfermeras realizaron 52 115 observaciones de higiene de las manos entre 2013 y 2018. El cumplimiento anual de la higiene de las manos aumentó significativamente del 76,4 % (2762/3617) en 2013 al 88,5 % (9034/10 211) en 2018 (*P* < 0,0001). Durante el mismo tiempo, el número de infecciones relacionadas con la atención médica disminuyó de 2012 a 1831, y su incidencia por cada 1000 días de pacientes se redujo de 14,0 a 11,7 (*P* < 0,0001). Se encontró una correlación negativa débil pero estadísticamente significativa entre la incidencia mensual de las infecciones relacionadas con la atención médica y el cumplimiento de la higiene de las manos (r = -0,48; *P* < 0,001).

**Conclusión:**

El cumplimiento de las prácticas de higiene de las manos que realizan los médicos y las enfermeras mejoró con la observación directa y la retroalimentación, lo que se asoció a una disminución de la incidencia de las infecciones relacionadas con la atención médica. Se requieren más estudios para evaluar la contribución de la higiene de las manos a la reducción de las infecciones relacionadas con la atención médica.

## ملخص

الغرض تحديد التغيرات في الالتزام بنظافة اليدين بعد تطبيق الملاحظة المباشرة لممارسات نظافة اليدين لدى الأطباء والممرضات، وتقييم العلاقة بين التغيرات وحدوث حالات العدوى المرتبطة بالرعاية الصحية.

الطريقة قمنا بإجراء مسح تدقيقي داخلي في مستشفى رعاية فوق الثانوي في فنلندا خلال الفترة من 2013 إلى 2018. قام ممرضو مكافحة العدوى بملاحظة ممارسات نظافة اليدين بناءً على استراتيجية منظمة الصحة العالمية الخاصة بنظافة اليدين. قمنا باحتساب الالتزام بنظافة اليدين بعدد الملاحظات حيث تم ممارسة نظافة اليدين الضرورية، مقسومًا على إجمالي عدد الملاحظات حيث كانت هناك حاجة إلى نظافة اليدين. لقد قررنا حدوث حالات العدوى المرتبطة بالرعاية الصحية باستخدام برنامج مراقبة إلكتروني شبه آلي لحدوث العدوى. قمنا بحساب معامل ارتباط Pearson ( *r *) لتقييم العلاقة بين حدوث حالات العدوى المرتبطة بالرعاية الصحية، والالتزام بنظافة اليدين.

النتائج سجلت الممرضات 52115 ملاحظة بخصوص نظافة اليدين في الفترة ما بين عامي 2013 و2018. زاد الالتزام السنوي بنظافة اليدين بشكل ملحوظ من 76.4% (2762/3617) في عام 2013، إلى 88.5% (9034/10211) في عام 2018 (نسبة الاحتمال أقل من 0.0001). خلال نفس الفترة، انخفض عدد حالات العدوى المرتبطة بالرعاية الصحية من 2012 إلى 1831، وانخفض معدل حدوثها لكل 1000 مريض/يوم من 14.0 إلى 11.7 (نسبة الاحتمال أقل من 0.0001). لقد اكتشفنا علاقة ضعيفة، ولكنها ذات دلالة إحصائية سلبية، بين المعدل الشهري لحدوث العدوى المرتبطة بالرعاية الصحية، والالتزام بنظافة اليدين (معامل الارتباط r = -0.48؛ نسبة الاحتمال أقل من 0.001).

الاستنتاج تحسن التزام الأطباء والممرضات بممارسات نظافة اليدين مع الملاحظة المباشرة والتقييم، وارتبط هذا التغيير بانخفاض في حدوث حالات العدوى المرتبطة بالرعاية الصحية. هناك حاجة إلى مزيد من الدراسات لتقييم مساهمة نظافة اليدين في الحد من حالات العدوى المرتبطة بالرعاية الصحية. ****

## 摘要

**目的:**

在直接观察医生和护士的手部卫生习惯之后确定手部卫生合规性的变化，并且评估这些变化与卫生保健相关性感染发生率之间的关系。

**方法:**

从 2013 年至 2018 年，我们在芬兰的一家三级医院进行了内部审核调查。感染控制专科护士根据世界卫生组织的手部卫生策略观察了手部卫生习惯。我们计算手部卫生合规性的方法为：采取了必要手部卫生措施的观察次数除以需要保持手部卫生的观察总数。我们使用半自动化的电子发生率监测程序来确定卫生保健相关性感染的发生率。我们计算了皮尔逊相关系数 (*r*)，以评估卫生保健相关性感染发生率与手部卫生合规性之间的关系。

**结果:**

在 2013 年至 2018 年之间，护士进行了 52,115 次手部卫生观察。年度手部卫生合规性从 2013 年的 76.4％ (2762/3617) 显著提升至 2018 年的 88.5％ (9034/10 211) (*P* < 0.0001)。同时，卫生保健相关性感染数量从 2012 例降至 1831 例，观察对象每 1000 个入院日的发生率从 14.0 降至 11.7 (*P* < 0.0001)。我们发现每月卫生保健相关性感染的发生率与手部卫生合规性之间存在微弱但在统计学上呈显著的负相关性 (r = −0.48; *P* < 0.001)。

**结论:**

通过直接观察和反馈改善了医生与护士在手部卫生习惯方面的合规性，并且这种变化与医疗保健相关性感染的发生率降低相关。需要进一步的研究来评估手部卫生对减少卫生保健相关性感染的有利因素。

## Резюме

**Цель:**

Выявить изменения в соблюдении правил гигиены рук с момента введения непосредственного наблюдения за гигиеной рук для врачей и медицинских сестер и оценить взаимосвязь между данными изменениями и заболеваемостью внутрибольничными инфекциями.

**Методы:**

Авторы проводили опросы во время внутренних проверок в больнице третичной медицинской помощи в Финляндии в период с 2013 по 2018 год. Медицинские сестры отделения инфекционного контроля наблюдали за осуществлением на практике гигиены рук в соответствии с правилами, определенными Всемирной организацией здравоохранения. Авторы рассчитали коэффициент соответствия требованиям гигиены рук как соотношение количества наблюдений, в ходе которых правила гигиены рук соблюдались, к общему количеству наблюдений, в ходе которых соблюдение таких правил было необходимо. Авторы также определили показатель заболеваемости внутрибольничными инфекциями с применением полуавтоматической программы электронного надзора за заболеваемостью. Был рассчитан коэффициент корреляции Пирсона (*r*) для оценки взаимосвязи между заболеваемостью внутрибольничными инфекциями и соблюдением правил гигиены рук.

**Результаты:**

В период с 2013 по 2018 год медицинские сестры выполнили 52 115 наблюдений за гигиеной рук. Ежегодный показатель соблюдения правил гигиены рук значительно вырос: с 76,4% (2762 случая из 3617) в 2013 году до 88,5% (9034 из 10 211 случаев) в 2018 году (*P* < 0,0001). В тот же период количество случаев внутрибольничных инфекций сократилось с 2012 до 1831, а заболеваемость на 1000 пациенто-дней упала с 14,0 до 11,7 (*P* < 0,0001). Авторы обнаружили слабую, но статистически значимую отрицательную корреляцию между ежемесячной заболеваемостью внутрибольничными инфекциями и соблюдением правил гигиены рук (r = –0,48; *P* < 0,001).

**Вывод:**

Соблюдение врачами и медицинскими сестрами правил гигиены рук улучшилось с момента введения прямого наблюдения и отчетов о результатах, и это изменение связано со снижением показателей заболеваемости внутрибольничными инфекциями. Для оценки роли гигиены рук в снижении заболеваемости внутрибольничными инфекциями необходимо проведение дальнейших исследований.

## Introduction

Evidence shows that improving hand-hygiene practices reduces the frequency of health-care-associated infections in hospitals.^1^^,^^2^ Studies have examined the association between hand-hygiene and health-care-associated infections: some were conducted over relatively short periods,^3^^,^^4^ some focused on specific infections, such as central-line-associated infections or *Staphylococcus aureus* bacteraemia,^5^^–^^11^ and some relied on mathematical models to predict the incidence of such infections.^3^^,^^9^

Several studies have demonstrated improvements in hand-hygiene compliance after implementation of interventions to promote hand hygiene,^3^^,^^9^^–^^11^ but which individual measures are most effective or how to maintain long-term improvements is still unknown.^12^ Despite promising new electronic systems for automatic monitoring of hand-hygiene compliance, these systems have notable limitations in real-world settings; in particular, they are expensive and require special methods, such as wireless technology.^13^^–^^15^ These systems also typically only provide data on hand-hygiene compliance when entering or leaving patient rooms. In addition, very little evidence exists on the effectiveness of hand-hygiene improvements at reducing the incidence of health-care-associated infectionss.^12^^,^^15^^,^^16^

We conducted a 6-year hospital-wide internal audit survey based on the World Health Organization’s (WHO) model for hand hygiene^1^ to evaluate hand-hygiene practices by direct observation by trained observers and provide immediate feedback. This method is considered the gold standard for monitoring hand-hygiene performance.^1^^,^^17^ At the same time, we recorded the incidence of health-care-associated infections across the hospital using a semi-automated electronic incidence surveillance programme. We hypothesized that changes in hand-hygiene compliance observed during our survey would be reflected in the incidence of health-care-associated infections.

## Methods

### Study design and setting

This longitudinal internal audit survey was conducted in Oulu University Hospital (OYS), a tertiary-care teaching hospital in northern Finland, between January 2013 and December 2018. This hospital has 792 beds and provided 223 559 patient days of care in 2018.

### Hand hygiene in the hospital

The hospital’s infection control department had used various methods to improve hand hygiene in the two decades before this survey. For example, in May 2010, the department started a new campaign based on WHO’s hand-hygiene improvement strategy.^1^ The hospital had previously implemented elements of this strategy, namely system change, education for health-care workers, evaluation and feedback, reminders in the workplace, and an institutional safety climate. As a result, alcohol-based handrub formulations have been available at the point of care (with bottles and mounted dispensers in every patient room or bay, and on every bedhead) since the early 1990s. In addition, the hospital established a network of infection-control link nurses in the late 1990s to improve infection control practices in their wards, for whom regular education and group meetings were held six to eight times a year. The infection control team provides regular training for health-care workers (including new staff and students) on proper hand hygiene. The hospital’s intranet site has held records of the annual consumption of alcohol-based handrub per 1000 patient-days in different wards since 1997 and of the annual health-care-associated infection rates per 1000 patient-days since 2008. Over the years, the hospital has placed various reminders about hand hygiene in patient-care units and staff areas. In 2010, the hospital hung paintings on the walls of the hospital entrance encouraging patients to remind health-care workers to use handrub. Infection prevention, and hand hygiene in particular, have been components of the hospital’s patient-safety strategy since 2013.

The hospital uses alcohol-based handrubs containing 70% (volume/volume) ethanol (tested according to European Standard, EN 1500)^18^ for hand hygiene in wards and outpatient clinics. The hospital prohibits the wearing of watches, hand jewellery and artificial nails during patient care.

### Observation of hand-hygiene

In January 2013, infection control link nurses began regular direct observation of hand-hygiene compliance at inpatient and outpatient locations to improve hand-hygiene compliance in health-care workers at the hospital.^19^ All doctors and nurses treating patients at the hospital were the study population. The link nurses informed doctors and nurses when they were making observations of hand-hygiene compliance and explained that the observations were part of the hospital’s quality evaluation process. In addition, the link nurses explained to patients that the observations were only on staff behaviour with the aim of improving professional practices.

The link nurses recorded information on the following variables during each observation: (i) duration of hand rubbing (in seconds); (ii) the observed moment according the WHO strategy (before touching a patient, before a clean or aseptic procedure, after touching a patient, after risk of exposure to a bodily fluid and after touching patient surroundings);^1^ (iii) profession of the person observed (doctor or nurse); and (iv) the ward where the observation was made. The target number of observations was at least 10 observations per ward a month. This observation and reporting exercise requires about 4–6 working hours for each ward per three-week cycle.

Between 2013 and 2016, the link nurses recorded hand-hygiene observations on paper (using stopwatches to measure timings) and transferred the data to an Excel spreadsheet (Microsoft, Redmond, United States of America). To reduce the time required to perform direct observations, the hospital’s infection control unit, OYS TestLab, which offers a systematic approach to enable and support the development of health and medical technology products at Oulu University Hospital, and FCG Flowmedik Oy (Helsinki, Finland) developed a mobile device (the web-based eRub-tool) that facilitates the coding of observations. Therefore, since 2017, the link nurses have made their observations using this device. The reports on the total number of hand-hygiene observations and the type of hand-hygiene moment are made available immediately after the observation on the hospital’s intranet. Data gathered between 2013 and 2016 were also transferred to the eRub-tool.

The implementation costs of this study were negligible because the hospital had established the link nurse programme several years before this survey began. Infection-control link nurses spent one working day every 3 weeks observing hand hygiene and checking all registered antibiotic prescriptions following patient discharge. Since our hospital was a pilot hospital where the web-based eRub-tool was developed, the use of this system was free of charge for our hospital.

### Variables studied

#### Use of handrubs

We obtained data on the yearly use of alcohol-based handrub in litres per 1000 patient-days from the hospital’s financial records. We determined the number of 1000 patient-days per month or per year by summing the corresponding number of overnight stays made by patients at the hospital and dividing the total by 1000.

#### Hand-hygiene compliance

We calculated hand-hygiene compliance as the number of observations where the necessary hand hygiene was practised divided by the total number of observations where hand hygiene was needed. We also extracted the duration of hand rubbing as recorded in the eRub database. We calculated compliance with the hand-rubbing time on a monthly basis over the 6-year study period. The length of hand rubbing recommended by WHO is 20–30 s.^1^ Depending on the size of hands we recommended one or two measures of handrub from the dispenser; so the approximate volume of handrub was 1.6 mL or 3.2 mL).

At the individual level, the link nurses gave verbal feedback on hand-hygiene performance immediately after the hand-hygiene observation. At the group level, feedback was given during regular ward meetings to the personnel on the wards by the link nurses and infection control nurses. At the organization level, the hand-hygiene compliance results were available to all staff of the hospital who have access to the hospital’s intranet.

#### Health-care-associated infections

We determined the incidence of health-care-associated infections by analysing the hospital’s records. The hospital uses a semi-automated electronic incidence surveillance programme that is linked to all the electronic databases of the hospital.^20^ When an antibiotic is added to the patient’s prescription, the programme automatically opens an enquiry form that doctors have to complete. Doctors are required to indicate whether the antibiotic was prescribed to treat a health-care-associated infection acquired in the hospital or a community-acquired infection. In each ward, two infection-control link nurses checked all of the registered antibiotic initiations during the hospital stay after patient discharge. In our hospital, health-care-associated infections are classified according to a modified version of the criteria proposed by the United States Centers for Diseases Control and Prevention.^21^ We calculated the incidence of health-care-associated infections per 1000 patient-days on a monthly or annual basis over the study period.

### Analyses

We did not calculate a sample size before the survey. Our goal was to obtain at least 50 000 hand-hygiene observations, which we considered sufficient for comparison of monthly hand-hygiene compliance figures with the incidence of health-care-associated infections. We used SAS, version 9.4 (SAS Institute, Cary, USA) for all analyses. We calculated the monthly change in incidence of health-care-associated infections (from 1 May 2013 to 31 December 2018) using a Poisson regression model. We give the results of the Poisson regression analysis as incidence rate ratio. We calculated the Pearson correlation coefficient (*r*) to evaluate the relationship between the incidence of health-care-associated infections and compliance with hand hygiene. For each year of the study, we calculated the mean monthly frequency of hand-hygiene compliance and 95% confidence interval (CI). We calculated the median and 25th and 75th centiles of rubbing times.

### Ethical considerations

The Hospital District Medical Director and Chief Nursing Officer of Oulu University Hospital approved this audit survey (registration number 246/2018). In Finland, the Medical Research Act (no. 488/1999) states that approval from the local ethics committee is not required for register-based surveys that do not process identifiable information. We consulted the secretary of the Regional Ethics Committee of the Hospital District who confirmed that our study was conducted in accordance with all applicable research regulations and norms of Finland.

## Results

Between May 2013 and December 2018, the link nurses made 52 115 observations where hand hygiene was needed (Table 1). In the past 3 years of the survey (2016–2018), these nurses made more than 10 000 observations each year. 

**Table 1:** Hand-hygiene observations and compliance, handrub use, and health-care-associated infections, Finland, 2013–2018

Year	No. of observations	No. of observations where compliance with hand hygiene was recorded	Hand-hygiene compliance, % (95% CI)	Litres of handrub used/ 1000 patient-days	No. of health-care-associated infections	No. of health-care-associated infections/ 1000 patient-days (95% CI)
2013	3617	2762	76.4 (74.8–77.6)	57	2 012	14.2 (13.4–14.9)
2014	7906	6479	82.0 (81.1–82.8)	68	2 003	13.6 (13.0–14.2)
2015	8599	7358	85.6 (84.8–86.3)	63	1 987	13.2 (12.7–13.8)
2016	11 346	9522	83.9 (83.2–84.6)	67	2 005	13.0 (12.4–13.6)
2017	10 436	9087	87.1 (86.4–87.7)	73	1 901	12.2 (11.6–12.7)
2018	10 211	9034	88.5 (87.8–89.1)	74	1 831	11.7 (11.2–12.2)
**Total**	**52 115**	**44 242**	**84.9 (84.6–85.2)**	**67**	**11 739**	**12.9 (12.7–13.1)**

CI: confidence interval.

**Fig. 1:**
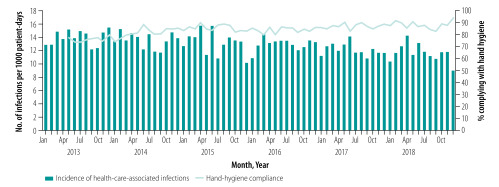
Monthly incidence of health-care-associated infections and hand-hygiene compliance, Finland, 2013–2018

The annual hand-hygiene compliance increased from 76.4% (2762/3617) to 88.5% (9034/10 211; *P* < 0·0001; Table 1) and the monthly hand-hygiene compliance increased from 77.5% (328/423) in May 2013 to 94.4% (456/483) in December 2018 (Table 2 and Fig. 1). The median hand-rubbing time over the 6 years was 21 s, with the 25th and 75th centile times being 13 s and 30 s, respectively. The consumption of alcohol-based handrub was 57 L per 1000 patient-days in 2013 and 74 L per 1000 patient-days in 2018. 

**Table 2:** Hand-hygiene observations and compliance, by month, Finland, 2013–2018

Month, year	Total no. of observations	Observations where compliance with hand hygiene recorded, no. (%)
May, 2013	423	328 (77.5)
June, 2013	347	257 (74.1)
July, 2013	426	314 (73.7)
August, 2013	496	376 (75.8)
September, 2013	527	406 (77.0)
October, 2013	563	440 (78.2)
November, 2013	493	367 (74.4)
December, 2013	342	274 (80.1)
January, 2014	297	220 (74.1)
February, 2014	908	701 (77.2)
March, 2014	731	585 (80.0)
April, 2014	677	544 (80.4)
May, 2014	763	621 (81.4)
June, 2014	516	457 (88.6)
July, 2014	436	366 (83.9)
August, 2014	599	483 (80.6)
September, 2014	828	670 (80.9)
October, 2014	823	699 (84.9)
November, 2014	905	771 (85.2)
December, 2014	423	362 (85.6)
January, 2015	460	383 (83.3)
February, 2015	468	406 (86.8)
March, 2015	598	507 (84.8)
April, 2015	701	634 (90.4)
May, 2015	988	837 (84.7)
June, 2015	637	534 (83.8)
July, 2015	494	438 (88.7)
August, 2015	815	726 (89.1)
September, 2015	725	640 (88.3)
October, 2015	893	734 (82.2)
November, 2015	742	621 (83.7)
December, 2015	1078	898 (83.3)
January, 2016	680	584 (85.9)
February, 2016	1351	1130 (83.6)
March, 2016	1028	823 (80.1)
April, 2016	816	709 (86.9)
May, 2016	1093	871 (79.7)
June, 2016	745	648 (87.0)
July, 2016	594	510 (85.9)
August, 2016	612	525 (85.8)
September, 2016	1005	828 (82.4)
October, 2016	1117	949 (85.0)
November, 2016	1295	1075 (83.0)
December, 2016	1010	870 (86.1)
January, 2017	746	641 (85.9)
February, 2017	982	836 (85.1)
March, 2017	1072	942 (87.9)
April, 2017	1189	1033 (86.9)
May, 2017	583	528 (90.6)
June, 2017	779	644 (82.7)
July, 2017	583	518 (88.9)
August, 2017	791	713 (90.1)
September, 2017	746	650 (87.1)
October, 2017	1003	854 (85.1)
November, 2017	1153	1009 (87.5)
December, 2017	809	719 (88.9)
January, 2018	1053	924 (87.7)
February, 2018	713	659 (92.4)
March, 2018	862	778 (90.3)
April, 2018	995	850 (85.4)
May, 2018	1157	1059 (91.5)
June, 2018	974	848 (87.1)
July, 2018	553	487 (88.1)
August, 2018	737	624 (84.7)
September, 2018	703	589 (83.8)
October, 2018	1037	927 (89.4)
November, 2018	944	833 (88.2)
December, 2018	483	456 (94.4)

**Fig. 2:**
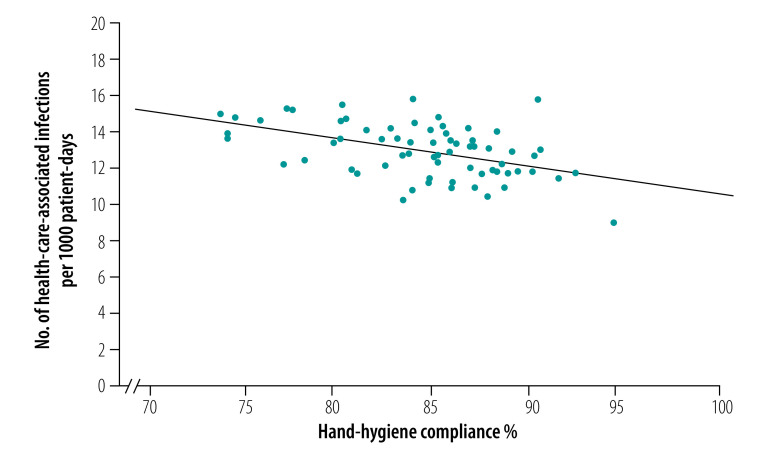
Correlation between monthly incidence of health-care-associated infections and hand-hygiene compliance, Finland, 2013–2018

The number of health-care-associated infections decreased from 2012 infections in 2013 to 1831 in 2018 and the incidence of health-care-associated infections decreased from 14.2 (95% CI: 13.4–14.9) to 11.7 (95% CI: 11.1–12.2) per 1000 patient-days (incidence rate ratio for monthly change = 0.999, *P* < 0.0001; Table 1 and Fig. 1). As shown in Fig. 2, we found a weak but statistically significant negative correlation between the monthly incidence of health-care-associated infections and hand-hygiene compliance (*r* = −0.48; *P* < 0.001).

## Discussion

Our results show that during the 6-year period of regular hand-hygiene observations and immediate feedback, the frequency of annual hand-hygiene compliance of doctors and nurses improved from 76.4% in 2013 to 88.5% in 2018 (*P* < 0.0001). At the same time the hospital-wide incidence of health-care-associated infections decreased significantly.

Two previous studies have examined the association between hand-hygiene compliance and all hospital-wide health-care-associated infections. The first study showed that over a 4-year period when hand-hygiene improved from 47.6% (1349/2834) to 66.2% (1701/2569), the prevalence of health-care-associated infections decreased from 16.9% to 9.9%.^2^ The second study examined hand-hygiene compliance after implementation of an infection-control programme and health-care-associated infections in both general wards and an intensive care unit.^4^ Over a 16-month follow-up period, hand-hygiene compliance increased from 41.0% (2235/5454) to 50.5% (3246/6428). Over the same time, the incidence of health-care-associated infections in the general ward was unchanged, but the number of severe health-care-associated infections in the intensive care unit decreased.^4^

In our study, when monthly hand-hygiene compliance was more than 80.0% over 2 years, the incidence of health-care-associated infections started to decrease (Fig. 2). In earlier studies where health-care-associated infections decreased, hand-hygiene compliance was at least 66.2% at the end of the study (number of opportunities = 2569 during the last observation period, December 1997)^2^ and the duration of the study period was at least 17 months.^2^^,^^5^^,^^6^^,^^9^^,^^22^ In a study with exceptionally high initial hand-hygiene compliance of 82.6%, compliance increased to 95.9% while the rate of health-care-associated infections fell by 6.0% during the 17-month study period.^22^ Further reductions in the incidence of health-care-associated infections at our hospital could therefore be possible if the annual hand-hygiene compliance rate at the hospital were raised above 90.0%.

We think that the increased compliance with hand hygiene is the most plausible explanation for the reduced incidence in health-care-associated infections in our study because the infection control practices applied at the hospital had remained largely unchanged in the years before the study. Although we cannot completely exclude the possibility that other factors may have contributed to the decline in health-care-associated infection rates, no other hospital-wide infection prevention strategies were introduced during the study period. However, the negative correlation between health-care-associated infections and hand-hygiene compliance was relatively weak (*r*^2^ = 0.23), suggesting that only 23% of the observed variation in the incidence of health-care-associated infections was related to changes in hand-hygiene practices. Therefore, some unidentified confounding factors could have contributed to the decrease in health-care-associated infections.

Previous reports have emphasized the need for multimodal approaches to achieve and sustain permanent improvements in hand-hygiene compliance.^1^ We believe that this is probably true when starting a hand-hygiene programme in a hospital with a low level of hand-hygiene compliance, as was demonstrated in an earlier hospital-wide compliance project.^2^ However, our results indicate that direct observation and immediate feedback on hand-hygiene procedures can induce a sustained increase in hand-hygiene compliance even when the annual compliance rate is relatively high to begin with (76.4% in our case). This relatively high initial compliance rate was a result of years of intensive efforts to improve hand-hygiene practices. Before this survey, four of the five components of the WHO’s multimodal hand-hygiene guideline^1^^,^^23^ had already been implemented at the hospital. However, this policy by itself did not reduce the incidence of health-care-associated infections to the levels observed after introducing regular observation and feedback. The important role of performance feedback in promoting and sustaining good hand-hygiene behaviour in hospital health-care workers has also been highlighted in previous reports.^24^

Direct observation makes it possible to assess compliance rates for all of the WHO hand-hygiene moments, and is seen as the gold standard for monitoring hand-hygiene compliance. However, direct observation is time- and resource-intensive, and can only be done in a small proportion of hand-hygiene opportunities.^14^ Furthermore, the risk of bias due to the Hawthorne effect can occur as health workers may improve their practices when under observation.^25^^,^^26^ Regardless of any possible Hawthorne effect, to improve your behaviour when you are observed, the infection-control link nurses made regular hand-hygiene observations in their own wards in a similar way over several years and gave feedback to their co-workers. We think that the Hawthorne effect might have had a positive influence on our results; it has earlier been shown that the Hawthorne effect can be used to encourage hand-hygiene compliance. In an ideal situation, the Hawthorne effect would be sustained with continuous observations improving hand-hygiene compliance and decreasing numbers of health-care-associated infections.^27^

Our study has several limitations. First, it is a non-randomized real-world internal audit survey conducted in a single university hospital in Finland. Further studies in other types of hospitals and countries will be needed to test the generalizability of these findings. Second, the link nurses only made hand-hygiene observations during the day shifts on week days, so the findings may not apply to night and weekend shifts. Third, our analysis is based only on hand-rubbing time, without the evaluation of rubbing technique. The observed median rubbing time of 21 s is acceptable because WHO recommends rubbing for 20–30 s.^1^ However, some of the staff observed did not spend enough time on rubbing because the 25th centile rubbing time was only 13 s. In addition, in the last year of the study (2018), no handrubbing was done at all in 11.5% (1177/10 211) of the occasions when hand hygiene was needed. Fourth, our survey did not assess whether the hand-hygiene technique was properly performed or if gloves were used. Fifth, we cannot rule out possible confounding factors (e.g. seasonal influenza, norovirus outbreaks in the community or holiday periods of health-care workers) that may have affected hand-hygiene compliance or the incidence of health-care-associated infections. Sixth, because we were interested only in hospital-level changes, we do not know which health-care-associated infections (e.g. surgical site infections or hospital-acquired pneumonia) decreased more after increased hand-hygiene compliance. We also did not compare hand-hygiene compliance between doctors and nurses or in different hospital wards.

A strength of this survey is the fact that observations of hand-hygiene compliance and health-care-associated infection surveillance were done regularly on a monthly basis over several years in a real-life setting. Health-care-associated infections were followed using a semi-automated electronic surveillance system.^20^ Implementation of electronic surveillance is considered a feasible way to identify health-care-associated infections.^28^ Importantly, the frequency of health-care-associated infections in patients discharged from the wards had remained steady in the 2 years before the study: the number of health-care-associated infections per discharged patient was 5.0% (1328/26 714) in 2011 and 5.0% (1266/25 457) in 2012.^20^ We were not able to obtain the number of discharged patients as in this earlier study, so could report incidence of health-care-associated infections per 1000 patients-days. These health-care-associated infections were recorded using the same semi-automated electronic surveillance system used during the survey. The introduction of the eRub-tool made easier to observe hand-hygiene and saved the link nurses time. Similarly, positive outcomes have been achieved using mobile tools for hand-hygiene observation in other studies.^5^^,^^29^

Further studies are needed to evaluate the contribution of hand-hygiene techniques to reducing the incidence of health-care-associated infections and to determine which health-care-associated infections are most effectively prevented by improving hand-hygiene compliance.
